# The relationship between student interaction with generative artificial intelligence and learning achievement: serial mediating roles of self-efficacy and cognitive engagement

**DOI:** 10.3389/fpsyg.2023.1285392

**Published:** 2023-12-22

**Authors:** Jing Liang, Lili Wang, Jia Luo, Yufei Yan, Chao Fan

**Affiliations:** ^1^College of Management Science, Chengdu University of Technology, Chengdu, China; ^2^School of Logistics, Chengdu University of Information Technology, Chengdu, China; ^3^Business School, Chengdu University, Chengdu, China; ^4^Business School, Southwest Minzu University, Chengdu, China

**Keywords:** generative artificial intelligence (GAI), education, self-efficacy, cognitive engagement, learning achievement

## Abstract

Generative artificial intelligence (GAI) shocked the world with its unprecedented ability and raised significant tensions in the education field. Educators inevitably transition to an educational future that embraces GAI rather than shuns it. Understanding the mechanism between students interacting with GAI tools and their achievement is important for educators and schools, but relevant empirical evidence is relatively lacking. Due to the characteristics of personalization and real-time interactivity of GAI tools, we propose that the students–GAI interaction would affect their learning achievement through serial mediators of self-efficacy and cognitive engagement. Based on questionnaire surveys that include 389 participants as the objective, this study finds that: (1) in total, there is a significantly positive relationship between student–GAI interaction and learning achievement. (2) This positive relationship is mediated by self-efficacy, with a significant mediation effect value of 0.015. (3) Cognitive engagement also acts as a mediator in the mechanism between the student–GAI interaction and learning achievement, evidenced by a significant and relatively strong mediating effect value of 0.046. (4) Self-efficacy and cognitive engagement in series mediate this positive association, with a serial mediating effect value of 0.011, which is relatively small in comparison but also shows significance. In addition, the propensity score matching (PSM) method is applied to alleviate self-selection bias, reinforcing the validity of the results. The findings offer empirical evidence for the incorporation of GAI in teaching and learning.

## Introduction

Generative artificial intelligence (GAI) stands as a distinct and potent class of artificial intelligence. It generates human-like content based on deep learning models in response to diverse and complex commands and questions (Lim et al., 2023). One significant example is ChatGPT, which has garnered great attention for its impressive capabilities in generating human-like answers and responding in a wide array of languages. A tossed stone raises a thousand ripples. GAI tools spark debates about the role of traditional human efforts (Else, 2023; Stokel-Walker, 2023), and prompted ethical considerations like matters of originality and potential plagiarism (Lim et al., 2023; Yu, 2023).

In the field of education, GAI tools have demonstrated their unprecedented ability in many disciplines in a short time (e.g., Baidoo-Anu and Owusu Ansah, 2023; Dwivedi et al., 2023; Kieser et al., 2023; Peres et al., 2023). There are different attitudes towards the application of GAI among educators. While critics like Noam Chomsky (Open Culture, 2023) argue that GAI is “basically high-tech plagiarism” and “a way of avoiding learning,” many educators indicate that GAI could help improve instructional processes, such as personalized tutoring (Baidoo-Anu and Owusu Ansah, 2023; Kasneci et al., 2023), automated essay grading (Terwiesch, 2023), interactive learning (Kasneci et al., 2023), adaptive studying (Pedro et al., 2019), and producing multiple examples and explanations in teaching and learning (Mollick, 2023). Some education bodies, such as New York City public schools (Lukpat, 2023), announced the ban on the use of ChatGPT. However, prohibiting GAI tools may have harmful effects such as the Streisand Effect (Jansen and Martin, 2015) and psychological resistance (Brehm, 1989). The former makes GAI more popular when banned, and the latter triggers student resistance to the rules (Brehm, 1989). Promoting an understanding of GAI technologies, instructing students on beneficial engagement with these tools, and openly debating their merits and drawbacks present a more enduring solution than simply prohibiting their use (Kasneci et al., 2023). Peres et al. (2023) also claim that our education should prepare students for their jobs after graduation, including mastering how to use these up-to-date tools.

On this basis, this paper takes an open and inclusive attitude towards the application of GAI, exploring the relationship between student–GAI interaction and their learning achievement. Even though students may utilize GAI to produce abundant content, it does not guarantee high achievement for them. Although many theoretical studies have discussed this link (e.g., Baidoo-Anu and Owusu Ansah, 2023; Dwivedi et al., 2023; Lim et al., 2023), relevant empirical evidence still needs to be provided. The initial objective of this study is to furnish empirical evidence concerning the association between student–GAI interaction and students’ learning achievement.

In addition, this paper investigates through which path the student–GAI interaction level links to their learning achievement. On the one hand, since GAI tools are easy to access (e.g., ChatGPT, driven by GPT3.5, is available for free), it is equivalent to providing users with multi-domain and executive-capable personal assistants at very low costs. With these powerful “personal assistants,” students may argue that they can solve tougher problems and complete more difficult tasks, that is, students could have a higher level of self-efficacy through the interaction with GAI. On the other hand, the real-time interactivity and instant feedback of GAI could motivate students to be more actively involved in learning tasks. Interaction with GAI is not limited by time and location. If a student has questions about course study, interaction with the teacher may require an appointment, while interaction with ChatGPT can be done at any time. These real-time interactive processes of GAI increase cognitive engagement in learning for students (Bian et al., 2018; Asiri et al., 2021), and the increased cognitive engagement in-turn relates to higher learning achievement (Zhu et al., 2009; Sedaghat et al., 2011; Wang and Eccles, 2012; Pietarinen et al., 2014).

Furthermore, students possessing greater self-efficacy are likely to exhibit increased cognitive engagement (Linnenbrink and Pintrich, 2003; Walker et al., 2006). They are more likely to be interested in learning activities since they intend to believe they can succeed and are more willing to invest effort to explore and understand knowledge, thereby enhancing cognitive engagement. Therefore, we propose that self-efficacy and cognitive engagement in series mediate the mechanism between students–GAI interaction and their learning achievement.

The remainder of this paper is organized as follows: Section 2 introduces the theory and hypothesis development, section 3 describes the questionnaire participants, indicators, and scales, section 4 presents the regression models and bootstrap mediating effect test, and section 5 discusses the conclusions, implications, and limitation.

## Theoretical review and research hypothesis

### The relationship between student–GAI interaction and learning achievement

In the field of education, the extraordinary ability of GAI has attracted significant attention among educators. The advantages of GAI include but are not limited to promoting personalized and interactive learning (Kasneci et al., 2023), providing quick feedback (Baidoo-Anu and Owusu Ansah, 2023), and generating prompts for formative assessment activities (Dijkstra et al., 2023; Terwiesch, 2023).

Interactive theory is usually used to describe the interaction between human and human, human and machine, as well as human and environment (Zhou and Wei, 2010; Freeman and Ambady, 2011). According to Interactive Theory, the feedback that individuals receive during the interaction process is important (Freeman and Ambady, 2011). GAI can provide real-time and personalized feedback (Baidoo-Anu and Owusu Ansah, 2023; Kasneci et al., 2023), which helps students to have a more accurate assessment of their strengths and weaknesses, thus making targeted improvements. Besides, GAI can dynamically adjust instructional content and methods according to the needs and reactions of each student, which is consistent with the viewpoint in Interactive Theory that effective interaction must be “bidirectional” and “dynamic” (Freeman and Ambady, 2011; Nowland et al., 2018). Compared with interacting with teachers, GAI could provide instant and continuous interaction without being limited by time and location. Compared with traditional educational tools such as books and exercises, GAI might be more interactive and can increase students’ cognitive engagement through gamification and incentive mechanisms. On the other hand, GAI could provide rich educational resources and diverse learning methods (learning games, videos, tests, simulation experiments, etc.), helping students carry out effective learning, memory, and a general understanding of reasoning, thereby enhancing and consolidating metacognitive knowledge for students (Vrugt and Oort, 2008; Azevedo, 2020). Following these lines, we propose that GAI is conducive to improving learning achievement and bring up Hypothesis 1:

*Hypothesis 1*: There is a positive relationship between student–GAI interaction and their learning achievement.

### The mediating role of self-efficacy

Self-efficacy theory explains the level of confidence an individual develops on a particular task (Bandura, 1997), which includes an individual’s assessment of their own ability to achieve the goal and their confidence in achieving it. Self-efficacy is gradually formed through individual experience, observation, and interaction. In educational contexts, students with higher self-efficacy tend to lead to higher academic achievement because they believe they can achieve their goals (Yokoyama, 2019).

This study indicates that GAI tools could serve as powerful assistants for students, leading to their increased self-efficacy. First, GAI has incredible capabilities to perform complex tasks such as writing articles (O’Connor, 2022), stories, poems, essays (Lucy and Bamman, 2021), images (Reed et al., 2023), providing textual summaries or extensions or even writing and debugging raw computer code (Kalliamvakou, 2022; Tate et al., 2023). In the interacting process, GAI tools demonstrated executive force and creativity that are unimaginable by humans (Baidoo-Anu and Owusu Ansah, 2023). Through interacting with these technologies, students can realize they may use GAI tools to create brilliant and satisfying content or solve more difficult tasks. They may have a higher assessment of their ability to achieve goals with the assistance of GAI. That is, student–GAI interaction could improve students’ self-efficacy.

Second, GAI can generate content based on students’ understanding level and subject background, providing a personalized learning experience. Students’ knowledge backgrounds are different in varied subjects. For those concepts that are abstract or completely foreign, they may need multiple explanations and cases to understand them (Ericsson and Pool, 2016). Creating multiple interpretations of a concept is a complex and time-consuming task for the instructor (Mollick, 2023). Tailoring explanations to students’ learning levels also requires the instructor to pay close attention to new trends and students’ cognitive loads (Lim et al., 2023). With limited time and energy, it may not be possible for instructors to take into account every student with their diverse needs, while GAI tools can help to improve this situation (Mollick, 2023). When interacting with AI, students can feel that they are in a tailor-made and personalized learning environment (Mollick, 2023), and can access and understand complex knowledge more easily (Peres et al., 2023). Thus, students might be more confident in learning, manifested by a higher level of self-efficacy.

On the other hand, higher self-efficacy links to improved academic performance has been extensively studied in the existing educational literature (Schunk, 1995; Robbins et al., 2004; Cassidy, 2015; Honicke and Broadbent, 2016; Doménech-Betoret et al., 2017; Yokoyama, 2019). Self-efficacy has been proven to affect students’ effort, persistence, interest, and achievement in learning activities (Schunk, 1995). Students with higher self-efficacy were more engaged, worked harder, persisted longer, showed greater interest in learning, and achieved higher grades (Schunk, 1995; Robbins et al., 2004; Yokoyama, 2019). Based on the above discussion, we hypothesize:

*Hypothesis 2*: The positive relationship between student–GAI interaction and learning achievement is mediated by students’ self-efficacy.

### The mediating role of cognitive engagement

From a constructivist perspective, cognitive engagement in learning is the extent to which students mentally invest in their learning activities, such as applying knowledge and cognitive tactics to accomplish the task (Chapman, 2002). Unlike physical engagement, cognitive engagement focuses more on mental activities such as thinking, planning, problem-solving, and decision-making (Greene, 2015). In educational settings, cognitive engagement is often used to describe how active students are in a course learning and whether they are actively thinking, solving problems, and interacting with material and massive information. The higher level of cognitive engagement students exhibit in the learning process can result in better related academic outcomes (Zhu et al., 2009; Sedaghat et al., 2011; Wang and Eccles, 2012; Pietarinen et al., 2014).

Based on Interactive Theory, we argue that the degree to which students interact with GAI affects students’ cognitive engagement and thus relates to learning achievement. The Interactive Theory views the feedback individuals receive during the interaction process to be very important (Hyland and Hyland, 2019). GAI tools’ real-time interactivity and instant feedback can motivate students to engage more actively in learning. GAI can also provide abundant images, information and examples to stimulate students to think further and explore deeper. For example, the rich explanations generated by ChatGPT can attract interest and arouse the curiosity of students, thereby enhancing their cognitive engagement in the learning process.

More importantly, it should be noted that the content a GAI tool generates is strongly dependent on the quality and nature of the inputs provided to it (Chatterjee and Dethlefs, 2023; Terwiesch, 2023). For example, asking ChatGPT a specific question could return a decent answer, while without any specifics, the answers provided by ChatGPT seem terse and biased (Lim et al., 2023). Therefore, in order to get an accurate answer, students need to conduct multiple rounds of questions and constantly revise the wording of the questions. This process can enhance students’ cognitive engagement. In addition, ChatGPT may give fake or erroneous references and generate flaw-and-confident answers (Van Dis et al., 2023). Students cannot completely rely on these content without any thinking and doubt. They need to interact with existing materials to evaluate the quality of contents generated. This process of continuous interaction can subtly improve students’ cognitive engagement in learning. Hence, this study proposes:

*Hypothesis 3*: The positive relationship between student–GAI interaction and learning achievement is mediated by students’ cognitive engagement.

### The serial mediating role of self-efficacy and cognitive engagement

Existing studies have investigated the relationship between self-efficacy and cognitive engagement, indicating that higher self-efficacy leads to improved cognitive engagement (Linnenbrink and Pintrich, 2003; Walker et al., 2006). Students with higher self-efficacy are more willing to believe they can succeed and invest time and cognitive effort to explore and understand new knowledge. When encouraged by challenges, they are more confident in their ability to overcome difficulties and thus be more actively seeking strategies to solve problems. They also generally have a more persistent drive to learn. According to deep learning strategies, students with high self-efficacy are more inclined to adopt deep learning strategies such as thinking, analysis, and discussion. They are full of confidence in their abilities and believe that the time and energy they invest will be rewarded and bring success. So they are not stingy in investing cognitive efforts to deepen their learning and understanding of the content. To sum up, students with high self-efficacy are more inclined to maintain a high cognitive engagement in the learning process.

The multiple mediation model applies to a situation where there are multiple mediating variables between the independent and the dependent variables (Liu and Ling, 2009). The serial mediation model, one type of the multiple mediation models, applies to a situation in which multiple mediating variables also show relationships (e.g., Allen and Griffeth, 2001; Sun et al., 2022; Wang et al., 2022), while the parallel mediation model applies to the model that views multiple mediating effects as parallel effects (Niehoff, 2005). Based on motivation theory and self-efficacy theory, a student’s belief in ability and desire to participate in a particular activity will be positively related to his/her subsequent performance (Greene and Miller, 1996; Ryan and Deci, 2000). Additionally, empirical relationships have been found between student self-efficacy and cognitive engagement (Greene and Miller, 1996; Linnenbrink and Pintrich, 2003; Walker et al., 2006). Hence, we propose that self-efficacy and cognitive engagement could act as serial mediators in the relationship between the level of student–GAI interaction and learning achievement rather than parallel mediators. To elaborate, the serial relationship is: student–GAI interaction leads to increased self-efficacy, which then boosts cognitive engagement, and ultimately links to higher learning achievement. Combined with the previous discussion, we put forth Hypothesis 4 as follows:

*Hypothesis 4*: Students’ self-efficacy and cognitive engagement in series mediate the positive relationship between student–GAI interaction and learning achievement.

**Figure 1:**
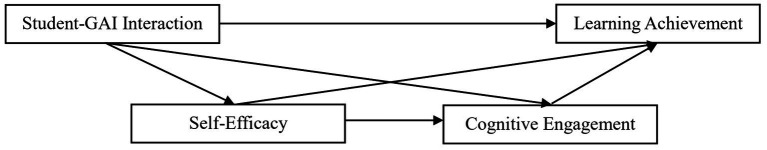
Hypothetical model of the serial mediating effect.

To sum up, our hypothetical model as shown in Figure 1.

## Materials and methods

### Participants

Data for this study was collected online via Wenjuanxing^1^, a web-based survey platform comparable to Mechanical Turk or Qualtrics, which is applied for conducting surveys in China. Wenjuanxing allows for nationwide responses and is widely utilized in research related to behavior and psychology (Sun et al., 2022). Based on this nationwide platform, our participants come from different grades, provinces, schools, and majors. Unlike undergraduates in other countries and regions who often be graded relying on essay writing, undergraduates in China are graded mostly relying on closed-book examinations^2^. These examinations usually require students to take closed-book tests in the class room without using any electronic products, which allays concerns that students use GAI to write essays and get false grades.

We set criteria in the participant recruitment phase, such as “participation in undergraduate education” and “over 18 years old,” so that the platform could collect responses that matched our research topic goals. Attention check questions (e.g., “Please indicate strongly disagree for this question.”) were included in the questionnaire to ensure respondents paid sufficient attention to each question (DeSimone et al., 2015). A total of 440 questionnaires were returned. Respondents who failed any attention check questions were excluded (28 out of 440). Due to the difficulty in guaranteeing the quality of the online surveys, we excluded responses that deviated by more than three standard deviations to prevent skewing from participants who may have selected the same answers throughout the questionnaire (23 out of 440). A total of 389 valid participants were obtained. Existing studies have shown that a valid sample size of 350–500 can proxy a target population of 5,000 or more (Sun et al., 2022), which indicates that our sample size of 389 is sufficient.

### Measurement

#### Student–GAI interaction scale

There are few empirical studies on the interaction between students and generative AI. We selected a scale from a similar study and reformulated it to match the subject of this study (Sun et al., 2022). For example, the original item, “Teacher–student interaction is getting longer in online education.” was reformulated as “Student–GAI interaction is getting longer in my course learning.” This scale has a total of 4 items. In addition to the example, others are “When I have questions during course learning, I use GAI to seek answers,” “I use GAI to ask for advice when doing course tasks,” and “My classmates are becoming more and more interested in GAI tools.” We use a 5-point Likert scale with scores ranging from 1 to 5, where 1 indicates strongly disagree, and 5 indicates strongly agree. We asked participants to recall experiences of using GAI during courses study and answer these questions. If the participant has no relevant experience interacting with GAI, the corresponding question can be filled in as 1 (strongly disagree). Improved scores on this scale indicate a greater degree of interaction between students and GAI, and the scores will be low if the student lacks experience interacting with GAI during the learning process. In order to optimize the content validity of the scale, we conducted a test in a small scope. The scale was discussed with 17 relevant researchers, experts, and teachers. They review the scale and comment on questions that are complex, unclear, or ambiguous. Based on their comments, the scale was adjusted to optimize content validity. The Cronbach’s alpha value for the student–GAI interaction is deemed acceptable: *α* = 0.974.

#### Cognitive engagement scale

The Cognitive Engagement Scale is derived from Wang et al. (2014) and Duncan and McKeachie (2005). We incorporate it into the setting of course learning to assess students’ cognitive engagement in course learning. For example, rewrite the original items “I try to figure out the hard parts on my own” and “I search for information from different places and think about how to put it together” (Wang et al., 2014) into “In course learning, I try to figure out the hard parts on my own” and “In course learning, I search for information from different places and think about how to put it together.” The self-efficacy scale has a total of 6 items. Students responded to these items on a 5-point Likert scale ranging from strongly disagree (1) to strongly agree (5). Cronbach’s alpha value for the Cognitive Engagement Scale is deemed acceptable: *α* = 0.713.

#### Self-efficacy scale

The Self-efficacy Scale is developed by Schwarzer and Jerusalem (1995) and Luszczynska et al. (2005) to assess students’ self-efficacy. Self-efficacy refers to the individual’s perception or belief about whether he can take adaptive behavior in the face of environmental challenges. This perception of “what can be done” reflects an individual’s sense of control over the environment. Therefore, self-efficacy is whether one can confidently view his/her ability to deal with various pressures. The self-efficacy scale has a total of 10 items (e.g., ‘I can always manage to solve difficult problems if I try hard enough.’) Students responded using a 5-point Likert scale anchored by 1 (strongly disagree) to 5 (strongly agree). Higher scores on this item indicate a higher degree of self-efficacy for each participant. Cronbach’s alpha value for the Self-efficacy Scale was deemed acceptable: *α* = 0.745.

#### Learning achievement

Learning achievement is measured by the difference between the 2023 academic year GPA and the 2022 academic year GPA for each participant. GPA is a commonly used academic performance evaluation index, which is used to measure the average grade level obtained by students within a semester or an academic year. In the first-round questionnaire, we asked the participants to report their 2022 academic year GPA.^3^ Since ChatGPT launched in November 2022, the 2022 academic year GPA can be considered a pre-test score. In the second-round questionnaire, we asked participants to report their 2023 academic year GPA, which can be considered as a post-test score. Some students may already have good grades, while others may already have poor grades. Therefore, considering the influence of interacting with GAI, we use the relative value (post-test score minus pre-test score) rather than the absolute value to measure students’ learning achievement. For comparability, we have performed dimensionless processing (i.e., *z*-score standardization) on variable values to eliminate the influence of dimension.

### Common method variance

Several methods were employed in this research to mitigate the risk of common method variance (Podsakoff et al., 2003). First, respondents were requested to complete surveys at two-time points 1 week apart. In the first round of questionnaires, we measured the independent variable (student–GAI interaction) and collected the GPA of the participants in the 2022 academic year and other individual characteristics. In the second round of questionnaires, we measured the mediator variable (self-efficacy and cognitive engagement) and collected the GPA of the participants in the 2023 academic year. Second, respondents were assured of anonymity and were uninformed about the specific objectives of the survey. Third, they were informed that there were no correct or incorrect responses and that their participation did not have any personal repercussions, encouraging honest answers. Fourth, the survey questions were presented in a random order. Last, we applied Harman’s single-factor test, and the results showed that the single-factor model explained 19.044% of the variance, which indicates that common method variance was not a concern in this study.

## Results

For the analysis of the questionnaire results, we use the bootstrap method to test the serial mediation effect of the indicators. The bootstrap method is a kind of non-parametric Monte Carlo method (Preacher and Hayes, 2004, 2008). Its essence is to re-sample the observation information, and then make statistical inferences on the overall distribution characteristics. Since this method makes full use of the given observation information, it does not require other assumptions of the model and adding new observations. Thus, the bootstrap method has the characteristics of robustness and high efficiency.

Compared with other statistical methods (e.g., regression analysis or structural equation modeling), the Bootstrap method has the advantage that it does not rely on the specified distribution assumption and is applicable to small sample sizes and complex models. In this study, we applied the bootstrap method to perform the serial mediation effect test using the PROCESS v4 macro test (proposed by Hayes, 2017) in SPSS 26.0.

### Descriptive and correlation analysis

Table 1 presents the demographic profiles of the respondents. Of the 389 respondents, 49.1% are male and 50.9% are female. 74.6% of respondents are between age 18 and 23. The number of respondents in the three professional types (skill, theory, and language) is relatively balanced.^4^

**Table 1:** Respondents’ profiles.

Variable	Category	Frequency	Percentage
Gender	Male	191	49.1%
	Female	198	50.9%
Age	18–20	117	30.1%
	21–23	173	44.5%
	24–26	57	14.7%
	27 or over	42	10.8%
Major type	Skill	141	36.2%
	Theory	130	33.4%
	Language	118	30.3%

Table 2 presents the means, standard deviations, and Pearson correlation coefficient for the main indicators. The results showed that learning achievement, student–GAI interaction, self-efficacy, and cognitive engagement are correlated at the 1% statistical significance level. Specifically, statistically significant correlations are observed between learning achievement and student–GAI interaction (*r* = 0.218, *p* < 0.01), learning achievement and self-efficacy (*r* = 0.314, *p* < 0.01), learning achievement and cognitive engagement (*r* = 0.419, *p* < 0.01). Student–GAI interaction and self-efficacy (*r* = 0.137, *p* < 0.01), student–GAI interaction and cognitive engagement (*r* = 0.271, *p* < 0.01), and self-efficacy and cognitive engagement (*r* = 0.434, *p* < 0.01).

**Table 2:** Descriptive statistics and Pearson correlation coefficient.

	Mean	S.D.	1	2	3	4
1. Learning Achievement	0.000	1.000	1.000			
2. Student–GAI Interaction	2.445	1.496	0.218**	1.000		
3. Self-Efficacy	3.611	0.420	0.314**	0.137**	1.000	
4. Cognitive Engagement	3.806	0.525	0.419**	0.271**	0.434**	1.000

** indicates that correlation is significant at the 0.01 level (two-tailed). The mean and standard deviation of learning achievement in the table are dimensionless, and the mean and standard deviation of the original data are 0.005 and 0.843.

### Analysis of serial mediating effect

Table 3 lists the estimates of the regression models: Model 1 estimates the relationship between student–GAI interaction and learning achievement; Model 2 estimates the relationship between student–GAI interaction and self-efficacy; Model 3 estimates the association of student–GAI interaction and self-efficacy with cognitive engagement; and Model 4 estimates the association of student–GAI interaction, self-efficacy, and cognitive engagement with learning achievement.

**Table 3:** Regression estimates.

Dependent variable	Model 1	Model 2	Model 3	Model 4
	Learning Achievement	Self-Efficacy	Cognitive Engagement	Learning Achievement
Student–GAI Interaction	0.146***	0.038***	0.069***	0.073**
	(4.391)	(2.717)	(4.510)	(2.336)
Self-Efficacy			0.431***	0.401***
			(7.869)	(3.442)
Cognitive Engagement				0.668***
				(6.641)
*R* ^2^	0.047	0.019	0.195	0. 219
*F*	19.282	7.382	46.867	36.015
*p* value	0.000	0.007	0.000	0.000

Numbers in parentheses are *t*-statistics value. Statistical significance is denoted by: ***p < 0.01, **p < 0.05, and *p < 0.1.

Hypothesis 1 posits that there is a significantly positive relationship between student–GAI interaction and their learning achievement. As seen in Model 1 in Table 3, the coefficient value for the student–GAI interaction level is found to be significant and positive (*β* = 0.146, *p* < 0.01), thus providing support for Hypothesis 1. Comparing estimates in Model 1 and Model 4, we find that the size and significance for the coefficient of the student–GAI interaction decreased (*β* changes from 0.146 to 0.073, and statistical significance changes from 0.01 to 0.05) after the mediating indicators are included. This indicates that self-efficacy and cognitive engagement mediate part of the relationship between student–GAI interaction and learning achievement.

Regarding self-efficacy, estimates in Model 2 show that student–GAI interaction positively relates to self-efficacy at the 1% statistical significance level (*β* = 0.038, *p* < 0.01), and Model 4 indicates that self-efficacy positively relates to learning achievement (*β* = 0.401, *p* < 0.01), which provide support for Hypothesis 2. With respect to cognitive engagement, results in Model 3 indicate that student–GAI interaction positively relates to cognitive engagement at the 1% statistical significance level (*β* = 0.069, *p* < 0.01), and Model 4 shows that cognitive engagement positively relates to learning achievement (*β* = 0.668, *p* < 0.01), which supports Hypothesis 3. Moreover, estimates in Model 3 also show that higher self-efficacy is linked to increased cognitive engagement (*β* = 0.431, *p* < 0.01). The above results provide support for Hypothesis 4.

We further analyzed the size and significance of each mediation by the Bootstrap method. Confidence intervals for indirect effects were calculated by Bootstrap repeated sampling to determine the statistical significance of mediating effects. The results are shown in Table 4.

**Table 4:** Bootstrap results for the mediation effect.

Mediating path	Indirect effect	Boot standard error	*p* value	95% confidence interval		Relative mediation effect	Total mediation effect
				Lower limit	Upper limit		
Total Effect	0.1456	–	0.000	0.0804	0.2108	–	100.00%
Total Indirect Effect	0.0728	0.0161	–	0.0429	0.1068	100.00%	50.00%
Indirect Effect 1	0.0154	0.0073	–	0.0029	0.0312	21.15%	10.58%
Indirect Effect 2	0.0463	0.0126	–	0.0237	0.0736	63.60%	31.80%
Indirect Effect 3	0.0111	0.0049	–	0.0028	0.0222	15.25%	7.62%

Level of confidence for all confidence intervals in output is 95%. Number of bootstrap samples for percentile bootstrap confidence intervals is 5000. Mediating path in this table are: Indirect effect 1 represents “Student–GAI Interaction➔Self-Efficacy➔Learning Achievement”; Indirect effect 2 represents “Student–GAI Interaction➔Cognitive Engagement➔Learning Achievement.” Indirect effect 3 represents “Student–GAI Interaction➔Self-Efficacy➔Cognitive Engagement➔Learning Achievement.”

Table 4 reveals that the Bootstrap 95% confidence interval for the mediation roles of both self-efficacy and cognitive engagement does not include zero (both the lower and upper limits exceed zero). This confirms that self-efficacy and cognitive engagement significantly mediate part of the relationship between student–GAI interaction and learning achievement. The value of the total indirect effect is 0.0728, which is primarily achieved through three pathways: (1) indirect effect 1 (0.0154): student–GAI interaction➔self-efficacy➔learning achievement; (2) indirect effect 2 (0.0463): student–GAI interaction➔cognitive engagement➔learning achievement; and (3) indirect effect 3 (0.0111): student–GAI interaction➔self-efficacy➔cognitive engagement➔learning achievement. Indirect effect 1, indirect effect 2, and indirect effect 3 accounted for 10.58, 31.80, and 7.62% of the total effect, respectively. The bootstrapping results further support that self-efficacy and cognitive engagement act as separate mediators and also jointly act as serial mediators between the student–GAI interaction level and learning achievement for students.

**Figure 2:**
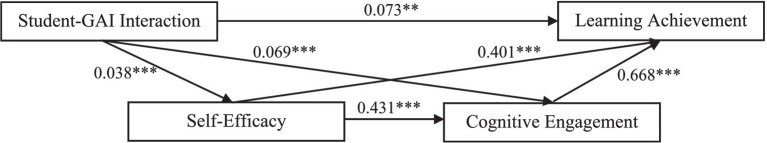
The serial mediation model of the impact of student–GAI interaction level on learning achievement. *** indicates *p* < 0.01 and ** indicates *p* < 0.05.

We summarize these results as Figure 2.

### Self-selection bias

There may be self-selection bias concerns in the mediating models. For example, there may be differences in the characteristics of students interacting with GAI tools or not. These differences could lead to differences in their self-efficacy and cognitive engagement, that is, self-selection bias. To solve this issue, we use the propensity scores matching (PSM) technique to establish comparable sets for the treatment and control groups, and control the characteristic differences between the two groups. The basic idea of PSM is that for each treated individual, find one or more individuals who are not treated but are very similar to him/her in other observed characteristics (such as age, gender, major, etc.) (Rosenbaum and Rubin, 1983). These similar individuals are usually matched through propensity scores.

Specifically, in the context of course learning, the control group includes the participants who did not interact with GAI, indicated by $\;G_{i}=0$ , and the treatment group includes the participants who interacted with GAI, indicated by $\;G_{i}=1$ . 50.9% of the sample is in the control group and 49.1% of the sample is in the treat group. The output variable for the treatment group is expressed as $\;{\mathrm{Output}}_{i1}$ , and the control group is $\;{\mathrm{Output}}_{i0}$ . In our study, the output variables are self-efficacy and cognitive engagement. We focused on the different effects on participants’ output variables before and after interacting with GAI. This difference is known as the average treatment effect on the treated (ATT) (Liang et al., 2022):

$$\tau_{ATT}=E\left({\mathrm{Output}}_{i1}|\;G_{i}=1\right)-E\left({\mathrm{Output}}_{i0}|\;G_{i}=1\right)$$

where $E\left({\mathrm{Output}}_{i1}|\;G_{i}=1\right)$ denotes the average value of output variables for the participants that interacting with GAI, and $E\left({\mathrm{Output}}_{i0}|\;G_{i}=1\right)$ denotes the average value of output variables by assuming those participants did not interact with GAI. Since $E\left({\mathrm{Output}}_{i0}|\;G_{i}=1\right)$ is unobservable, we replace it by the average value for participants that did not interact with GAI in course learning, who have similar characteristics with the former, expressed as $E\left({\mathrm{Output}}_{i0}|\;G_{i}=0\right)$ . So the ATT can be computed as the difference in average of output variables between interacting and non-interacting with GAI participants:

$$\tau_{\mathrm{ATT}}=E\left({\mathrm{Output}}_{i1}|\;G_{i}=1\right)-E\left({\mathrm{Output}}_{i0}|\;G_{i}=0\right)$$

The equation builds on the condition that the characteristics of the participants in both treatment and control groups are the same. Following to Villalonga (2004), we set the propensity scores as the probability that the participant tends to interact with GAI on the vector of independent variables $\;X_{i}$ :

$$p\left(X_{i}\right)\equiv \mathrm{Pr}\left(G_{i}=1|X_{i}\right)=E\left(G_{i}|X_{i}\right)$$

where vector $X_{i}$ represents the characteristics that may also affect the output variables, including the participant’s age, gender, grade, province, major, senior high school subjects (Arts/Sciences), SAT score, 2022 academic-year GPA, student union activities and student awards. This information was collected from respondents in the first-round of the questionnaire.

**Figure 3:**
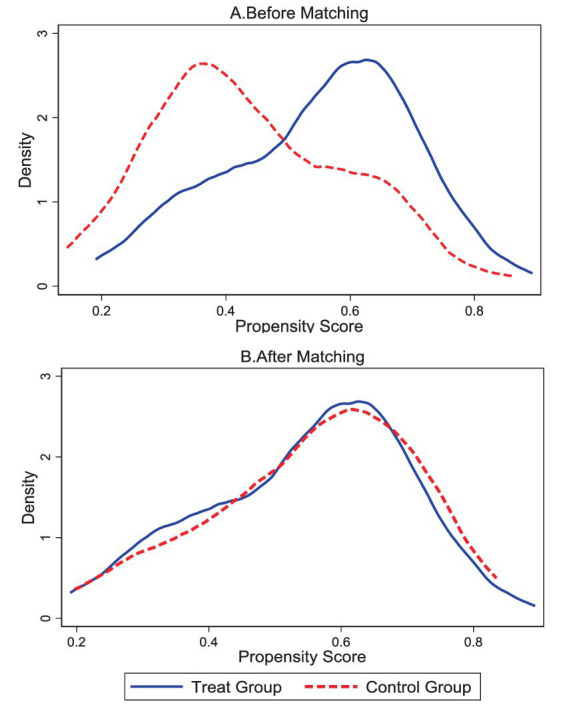
The graph of kernel density functions of treatment and control groups before and after matching.

Using the nearest neighbor matching method, we aligned the treatment and control sets and applied probit regression for estimating the propensity scores. The kernel density distributions for both the treatment and control groups are represented in Figure 3.

Figure 3A shows that there is a significant difference in the kernel density function between the treatment group (participants that interact with GAI) and the control group (participants that not interact with GAI) before matching. Figure 3B shows that the kernel density function images of the two groups are closer after matching, indicating that the treatment and control groups are more comparable in terms of these individual characteristics. Based on PSM, the treatment effect results for self-efficacy and cognitive engagement are summarized in Table 5.

**Table 5:** The PSM results.

Treatment variable	Output variable	Sample	Average value of output variable for the treatment group	Average value of output variable for the control group	ATT	*t*-stat
Interact with GAI	Self-Efficacy	Unmatched	3.6939	3.5110	0.1829^***^	3.45
		Matched	3.6934	3.5231	0.1703^**^	2.03
Interact with GAI	Cognitive Engagement	Unmatched	3.9333	3.7333	0.2000^***^	3.75
		Matched	3.9330	3.7160	0.2170^***^	2.72

50.9% of the sample is in the control group and 49.1% of the sample is in the treat group. Statistical significance is denoted by: ***p < 0.01, **p < 0.05, and *p < 0.1.

From Table 5, we can see that the average values of self-efficacy and cognitive engagement are significantly higher in the treatment group (participants that interact with GAI) than in the control group (participants that do not interact with GAI), and ATT values are significantly positive (ATT = 0.1829 for self-efficacy, and ATT = 0.2000 for cognitive engagement. |t| > 1.96). After the propensity score matching, the ATT values of self-efficacy and cognitive engagement are still statistically significant (ATT = 0. 1703 for self-efficacy, and ATT = 0. 2,170 for cognitive engagement. |t| > 1.96). The findings suggest that participants interacting with GAI would have higher levels of self-efficacy and cognitive engagement even after controlling the self-selection bias.

## Discussion

This study explores the relationship between students’ interaction with GAI and learning achievement, considering the mediating roles of self-efficacy and cognitive engagement. The empirical study collected responses of questionnaires from 389 participants. The results showed: (1) Overall, there is a significantly positive relationship between the degree of student–GAI interaction and their learning achievement. (2) This positive relationship is mediated by self-efficacy, with a significant mediation effect value of 0.015. (3) Cognitive engagement also acts as a mediator in the mechanism between the student–GAI interaction and learning achievement, evidenced by a significant and relatively strong mediating effect value of 0.046. (4) Self-efficacy and cognitive engagement in series mediate the positive relationship between the degree of student–GAI interaction and their learning achievement, with a serial mediating effect value of 0.011, which is relatively small in comparison but also shows significance.

### Theoretical implications

This study contributes to educational research in three ways. First, this study provided empirical evidence about the relationship between student–GAI interaction and their learning achievement, responding to the calls that require empirical insights to help us better understand the implications of GAI for education so as to build useful knowledge bases (Pedro et al., 2019; Lim et al., 2023; Peres et al., 2023). GAI tools have been mentioned as “strength enhancers” for instructors (Kasneci et al., 2023), helping to design instruction to accommodate students of different comprehension abilities and subject backgrounds, but applying GAI in teaching needs to be based on abundant empirical evidence (Mollick, 2023). Some scholars believe that using GAI tools can help students better understand the learning content and improve the mastery of knowledge, thereby enhancing their academic achievement (Baidoo-Anu and Owusu Ansah, 2023), while others argue that GAI may cause students to be lazy and over-dependent with no or little analytical abilities, thereby reducing the academic performance (Pedro et al., 2019; Lim et al., 2023). This study offers empirical support that the interaction between students and GAI is positively related to students’ academic performance, providing a reference for future incorporation of GAI in teaching.

Second, this study sheds light on the mediating roles of self-efficacy and cognitive engagement in the mechanism between the student–GAI interaction level and learning achievement. Extensive literature demonstrates that environment, actions, and behavior affect the psychological factors, which influence outcomes (e.g., Liang et al., 2021; Nagadeepa, 2021; Chhetri and Baniya, 2022; Wei et al., 2023). While many GAI-related studies have illustrated that GAI tools are characterized by personalization and interactivity (Baidoo-Anu and Owusu Ansah, 2023; Kasneci et al., 2023), it is unclear how these characteristics are associated with students’ psychological factors in course learning. This study indicates that students’ interaction with GAI positively relates to their self-efficacy and cognitive engagement in course learning, which enhances our understanding of the psychological channels through which interaction with GAI links to students’ learning.

Third, this study provides new insights into the mediating effects of self-efficacy and cognitive engagement in education by introducing interactions with novel technical tools as the independent variables. There is a considerable amount of research pointing out that self-efficacy and cognitive engagement play mediating roles in relation to students’ motivation, understanding, learning and achievements (Schunk and Pajares, 2001; Bandura, 2006; Van Dinther et al., 2011; Chong et al., 2018; Nagadeepa, 2021; Chhetri and Baniya, 2022; Wei et al., 2023). Following this line, we supplement empirical evidence that self-efficacy and cognitive engagement also mediate the association of students’ interaction with GAI tools and academic achievements and discuss their practical implications.

### Practical implications

#### The student–GAI interaction level affects learning achievement

This study finds a positive relationship between student–GAI interaction and their learning achievement during course learning. The results provide empirical support for the view that GAI positively relates to student learning performance, and we should take full advantage of GAI’s strengths rather than just banning it. We echo the opinion of Kasneci et al. (2023) and Mollick (2023) that if implemented carefully and thoughtfully in evidence-based teaching practices, artificial intelligence could be a “force multiplier” for teachers.

Based on this result, how to safely improve the level of interaction between students and GAI, so as to promote its impact on students’ learning achievement, is a concern in the education practices. First, like other technologies such as Python and MATLAB, instructors can introduce students to the correct way, steps, and precautions (Smaldino et al., 2006; Mollick, 2023) to use GAI tools, allowing students to use them to create text, images, audio, and other content according to course purposes, thereby stimulating the students–GAI interaction level. Second, problem-solving activities that require students to work with the GAI could be constructively designed (Oradee, 2012; Perera and Lankathilaka, 2023). For example, in a science class, let students work with GAI to analyze data, predict trends, and then come up with solutions together. Related assignments can require students to think deeply about the problem, analyzing different perspectives and possible solutions (Mousoulides et al., 2007). This encourages students to think independently, rather than simply relying on what the GAI provides. Third, encouraging students to be creative and expressive in their interactions with GAI. Let students know they can try out different ideas without fear of making mistakes. Related assignments can require students to express their ideas and come up with unique and creative solutions to ensure their work is original (Dorst and Cross, 2001). Last and importantly, we should establish a comprehensive evaluation system that includes interaction with GAI, so that students can clearly realize that the role of GAI is to assist, not completely replace their work. In the evaluation system, in order to avoid plagiarism and other moral issues caused by using GAI, more attention should be paid to creativity, in-depth thinking and analysis, problem-solving and creative process, ability improvement etc. (Liu et al., 2023; Yu, 2023). It requires more effort and attention in the future. We further call for dialogue among researchers, educators, and educational institutions on how to safely and constructively improve the student–GAI interaction level to support student learning.

#### The serial mediating role of self-efficacy and cognitive engagement

This study further sheds light on the mediating roles of self-efficacy and cognitive engagement in the mechanism between the student–GAI interaction level and learning achievement, and the degrees of mediating effects for different mediators vary. Among them, cognitive engagement serves as a mediator that has a greater effect on students’ learning achievement, followed by self-efficacy.

Based on these findings, we suggest that educators could pay primary attention to the effect on learners’ cognitive engagement when guiding them to interact with GAI. In the teaching design, instructors can consider designing challenging tasks that stimulate students’ interest and require deep thinking (Herft, 2023; Küchemann et al., 2023). These tasks can involve problem-solving, creative expression, or practical application and require students to interact with the GAI to obtain valuable outputs. Besides, exploratory tasks can also be designed to encourage students to explore the different functions and applications of GAI independently. Guiding students to discover the potential of GAI based on practical problems to increase their curiosity and initiative (Abdelghani et al., 2022). After the tasks are completed, students are encouraged to engage in reflection and discussion, sharing their experience of interacting with GAI, biases corrected, challenges encountered, and insights gained from it. This helps to increase the cognitive engagement of the learners.

The improvement of students’ self-efficacy can also be noticed in teaching practice interacting with GAI. Before a learning task begins, instructors could clearly state the goals and expectations of the task (Küchemann et al., 2023) so that students understand what they will achieve through their interaction with GAI. Clear goals help students develop self-confidence by knowing their efforts will be rewarded. When doing the task, prompt students to use the GAI to get more explanations and examples of concepts they do not understand. In addition, a feedback mechanism could be set during the interaction with GAI [see Herft (2023) and Jia et al. (2021) for more details to support and improve teaching assessment and feedback practices]. Students can know immediately whether their answers are correct. Timely feedback and recognition could also help to improve self-efficacy. After the assignment, share successful cases of interacting with GAI so that students can learn how others have achieved their academic goals by collaborating with GAI. This can stimulate students’ enthusiasm and self-confidence. Besides, review with the students their accomplishments and progress. Make them aware of their own growth and progress, thereby increasing their confidence in their abilities. By improving self-efficacy, students can develop positive learning attitudes, which is also conducive to improving cognitive engagement and enhancing learning achievement.

Although this research explores the relationship among student–GAI interaction, psychological variables and academic performance from the perspective of students, how to use these conclusions to improve education practices should start from the perspective of instructional design. Many instructional designs and techniques have proven valuable but are difficult to put into practice because they are time-consuming for overworked instructors (Kirschner et al., 2022; Mollick, 2023). Interacting with GAI could quickly and easily implement evidence-based instructional designs to provide guidance.

#### Research limitations and prospects

This study empirically tests the mechanism between student–GAI interaction and their learning achievement, and has value in both statistical and practical significance. However, it has several limitations. First, the participants in this study were Chinese, as Chinese universities typically use closed-book exams that do not use any electronic tools to evaluate students’ performance, but this may limit the generalizability of the findings. Future research may need to validate the conceptual model in other cultural contexts and countries to test how interactions with the GAI affect performance in different assessment modalities. Second, the relationship between the level of interaction with GAI and student learning achievement may also be affected by other factors, such as instructor ability, class atmosphere, school policies, etc. Hence, subsequent studies could investigate the specific conditions that moderate how interactions with GAI affect students’ academic performance from varied perspectives. Third, this study only carried out the questionnaire survey from the perspective of students. Future research can analyze the impact of interaction with GAI from the perspective of instructors, or pair instructors and students. Fourth, there are many types of university courses. This study does not distinguish between different disciplines to study the impact of GAI on teaching and learning. Future research could differentiate between varied disciplines and investigate the impact of the interaction with GAI on teaching and learning in that discipline. For example, considering the transformation brought by GAI, management discipline may pay more attention to decision-making, technical disciplines may pay more attention to the purpose of technology implementation, and music and art may pay more attention to originality. Fifth, future research can also consider a moderating effect model that considers students’ psychological factors as moderator variables and explores what psychological characteristics students possess are more susceptible to the impact of interaction with GAI tools. In addition, discussing the impact of students’ interactions with GAI tools in subdivided dimensions (e.g., the quality, single time, number of interactions, as well as the types and quantities of GAI tools etc.) is also worth further exploring. Last, studies could utilize longitudinal data to meticulously explore the causal pathways, providing further validation and deepening these preliminary findings.

## Data availability statement

The raw data supporting the conclusions of this article will be made available by the authors, without undue reservation.

## Ethics statement

The studies involving humans were approved by College of Management Science, Chengdu University of Technology. The studies were conducted in accordance with the local legislation and institutional requirements. Written informed consent for participation was not required from the participants or the participants’ legal guardians/next of kin because questionnaires were collected anonymously through a nationwide online platform, and participants’ names and other sensitive personal information were not identified. Participants were informed that there were no correct or incorrect responses and that their participation did not have any personal repercussions.

## Author contributions

JLi: Data curation, Formal analysis, Conceptualization, Methodology, Investigation, Funding acquisition, Writing – original draft. LW: Data curation, Funding acquisition, Investigation, Writing – original draft. JLu: Formal analysis, Methodology, Writing – review & editing. YY: Data curation, Funding acquisition, Investigation, Validation, Writing – review & editing. CF: Supervision, Project administration, Validation, Writing – review & editing.

## Footnotes

^1^Wenjuanxing website: https://www.wjx.cn, Available on Aug. 8, 2023.

^2^According to our investigation at a university in southwest China. Most courses (about 70%) graded for undergraduate students rely primarily on closed-book exams.

^3^The academic year in Chinese universities starts in September and ends in July of the following year, so the 2022 academic year GPA refers to the GPA score from September 2021 to July 2022. During that time period, GAI has not yet started to be widely used.

^4^Skills mainly represent engineering majors, such as machinery, materials, automation, computers, etc. Theory represents science and liberal arts majors, such as theoretical physics, mathematics, economics, management, philosophy, education, history, etc. Languages include English, German, French, Japanese, Spanish and other language majors.
